# Environmental exposures and cancer: using the precautionary principle*

**DOI:** 10.3332/ecancer.2019.ed91

**Published:** 2019-04-16

**Authors:** Lorenzo Cohen, Alison Jefferies

**Affiliations:** 1 Department of Palliative, Rehabilitation, and Integrative Medicine, Section of Integrative Medicine, The University of Texas MD Anderson Cancer Center Houston, TX 77030, USA; 2 Vital Matters, LLC, Houston, TX 77025, USA; * **Adapted from:** *Anticancer Living: Transform Your Life and Health with the Mix of Six* (Viking, 2018).

**Keywords:** cancer, environment, carcinogen, toxins, endocrine-disrupting compounds

## Abstract

Since the 1970s, more than 87,000 chemicals have been approved for commercial use. Yet of those thousands of chemicals, only just over one thousand have been formally examined and graded for their carcinogenic potential. Of those, five hundred have been found worthy of being graded on a cautiously worded scale ranging from “known” carcinogens to “possibly” carcinogenic. In addition to carcinogenic substances, a new field has emerged researching how environmental toxins cause endocrine or hormonal disruption. A class of these compounds known as endocrine-disrupting chemicals (EDCs) can be found in our food, our environment, and in the products we put on our bodies. Rather than being directly linked to causing cancer, like substances such as asbestos, EDCs influence our health by mimicking or enhancing or changing metabolic regulation. These compounds interfere with hormone production and metabolism in ways that may—especially over the long term—create biological conditions that make us more susceptible to cancer and other diseases. Most of us are exposed to a cocktail of environmental toxins on an ongoing daily basis and at a relatively low level of exposure. Given the lax regulation of chemicals and the reactionary approach of government regulators, it is up to the consumer to be diligent about reading labels and making healthy choices to limit exposure to chemicals and toxins. It is ideal to adopt the Precautionary Principle: until a chemical is found to be harmless, try to not use it. The precautionary principle means that you are maintaining awareness of what you are putting on and in your body and taking steps to avoid exposing yourself unnecessarily to toxins in your household and environment.

Since the 1970s, more than 87,000 chemicals have been approved for commercial use [1]. Yet of those thousands upon thousands of chemicals, only just over one thousand have been formally examined and graded for their carcinogenic potential [2]. Of that one thousand, fully five hundred have been found worthy of being graded on a cautiously worded scale: 120 chemicals have been identified as “known” carcinogens; another 81 have been identified as “probable” carcinogens; and another 299 as “possibly” carcinogenic, according to analysis published by the International Agency for Research on Cancer (IARC) [2], part of the World Health Organization (WHO).

But what about the other 86,600 (give or take) chemicals that are being inhaled, swallowed, or absorbed into the skin in an unfathomable number of combinations? How on earth do we capture data about these unseen substances and figure out if we need to be adding to the master list of known or possible carcinogens? What about the chemicals that may not be directly carcinogenic but may nonetheless modify our internal biology and play a role in the onset of serious diseases, including cancers?

We simply cannot escape our exposure to man-made chemicals. But there is a lot we can do to moderate our exposures. For the most part, it is up to us to become more conscious about the chemicals we are exposed to in the air we breathe, the water we drink, the furniture in our homes, the clothes we wear, and the products we put on our bodies every day. While onetime exposure to chemicals is often considered “safe,” many of these products are used every day, and the effects of long-term exposure, especially when combined with other chemicals, is largely unstudied and unknown. It is up to us to proceed through this chemically laden world we live in first and foremost with caution. In this regard, it is ideal to adopt the *Precautionary Principle*: until a chemical is found to be harmless, try to avoid exposure. The precautionary principle encourages maintaining awareness of what you are putting on and in your body and taking steps to avoid unnecessarily exposing yourself to toxins in your household and environment.

## Our homes are awash in chemicals

Toxic chemicals aren’t just to be found under our sinks. They’re in shampoos, body washes, lotions, hand sanitizers, perfumes, colognes, aftershave, and makeup, even our toothpastes and mouth rinses [3–5]. We simply cannot get away from them—unless we engage in a conscious, ongoing effort to identify them and find alternatives. Even then, it can be challenging, as chemical manufacturers have caught on to our wish to “go green” and they’ve become masterful at masquerading toxic products as being less harmful than they actually are. For instance, it turns out that “fragrance” is a mix of chemicals that don’t have to be disclosed by the manufacturer and often include endocrine-disrupting compounds that can affect our hormone levels over time [6]. Have you ever purchased kitchenware that says on the label “for decorative purposes only?” This means that there is likely lead or another toxic substance in the paint or finish and the bowl or dish should not be used for food storage or presentation [7, 8]. Glyphosate-based herbicides, like Roundup, that are used for weed control in our gardens have been classified as probably carcinogenic to humans by the WHO IARC [2]. Even that cash register receipt you have tucked into your pocket isn’t clean: it’s laced with bisphenol A (BPA; this is what makes the printed numbers adhere to the paper), a known endocrine disruptor, and research shows that you’ll show a spike of BPA in your system if you rub your face or eat right after handling a receipt [9, 10].

## Environmental toxins and endocrine disruption

Just in the last twenty years or so, a new field has emerged for researching how environmental toxins cause endocrine or hormonal disruption. Rather than being directly linked to the onset of specific cancers like the known fifty-four carcinogens identified by the EPA (such as asbestos), endocrine disrupters influence our health in different ways by mimicking or enhancing or changing metabolic regulation [11]. Most of us are exposed to a cocktail of environmental toxins on an ongoing daily basis and at a relatively low level of exposure. A class of these toxins known as Endocrine-disrupting chemicals (EDCs) can be found in our food, our environment, and in the products we put on our bodies. These compounds interfere with hormone production and metabolism in ways that may—especially over the long term—create biological conditions that make us more susceptible to cancer and other diseases [11, 12, 13]. 

Endocrine disrupters don’t work the way classical carcinogens work. Instead, they work like our natural hormone system, our endocrine system, at exquisitely low levels and affect the physiology of the body at different times of life—especially at times of development, which interferes with normal development. Carcinogens cause cellular damage and mutation, whereas endocrine disrupters cause ripples in overall development.

We see, for instance, early-onset puberty in both boys and girls when exposed to certain (though often different or uniquely combined) endocrine disrupters in the environment, in food, or in the consumer products these children use. A study by the U.S. Centers for Disease Control and Prevention found that girls exposed to high levels of a solvent used in toilet bowl deodorizers and air fresheners had their first menstrual period seven months earlier than those with lower exposure levels [14, 15]. That 2012 research is the culmination of a series of studies that suggest environmental toxins, especially those that mimic hormones, could be responsible for causing changes in humans. The chemical in this particular study, dichlorobenzene, is present in the body of nearly every person tested in the United States [16]. Meanwhile, research by Kushi et al. at Kaiser Permanente has been studying whether exposure to environmental chemicals leads to early puberty in more than twelve hundred prepubescent girls in three U.S. cities—New York, Cincinnati, and Oakland. After following the girls for a dozen years, Kushi and his team connected early breast development with two chemicals—triclosan, which is present in toothpaste and also in many other products, and 2,5-dichorophenol, a chemical used in pesticides and to chlorinate water [17, 18]. Those two chemicals were related to girls developing four to nine months sooner. A report in 2011 and 2012 found that boys in the United States were maturing between six months and two years sooner than they were in the 1970s [19, 20]. Researchers aren’t clear why this is happening. With girls, they blame estrogen-like chemicals in the environment, such as dichlorobenzene or BPA. But boys exposed to the same chemicals should have an opposite effect—delaying sexual maturation. One possible reason for early puberty in boys could be an increase in rates of obesity, which alter the body’s hormone levels. Unfortunately, the risk of both breast and prostate cancer increases with early onset puberty as higher lifetime estrogen and testosterone exposure is a risk factor for breast and prostate cancer, respectively [21–23].

There is also research suggesting a link between EDC and obesity and other metabolic conditions. A study in 2014 found that children exposed to chemicals used to soften plastic were more likely to be obese and at increased risk of diabetes [24]. Researchers at New York University examined the urine and blood of more than 750 adolescents and found increased insulin resistance, a precursor to diabetes, in teenagers with higher levels of di-2-ethylhexyl phthalate or DEHP [25]. Meanwhile, high levels of BPA were connected with being overweight or obese. The rates of obesity were twice as high in teens with the highest BPA levels compared to those with the lowest levels [26]. Researchers speculate that excess BPA could throw off hormonal balance and disrupt metabolism, but note that more studies are needed to show a direct link [27, 28].

What makes separating out the effects of different environmental carcinogens and EDCs so challenging is how fluidly and integrally they’re threaded throughout virtually every facet of our lifestyle. Janet Gray, PhD, a professor of neuroscience at Vassar College, has been studying endocrine disruptors for the past fifteen years. She explains that our natural hormone system helps maintain “homeostasis,” which is systemic hormonal balance. But, over time, endocrine disruptors and chemicals that mimic hormones can cause an imbalance in this delicate system [13]. “When this happens later in life, after our organs are fully-developed, we believe it may be influencing brain health issues and certainly the onset of cancers, such as breast cancer,” Gray explains. “When endocrine disruption happens to an embryo or a fetus in utero, the long-term consequences can be more dire. Additionally, we pass along this kind of endocrine disruption to future generations, which add a whole new element to the new field of epigenetics. For example, we have many studies now that show the correlation between women developing breast cancer now and the exposure of their female relatives decades earlier to DDT” (dichlorodiphenyltrichloroethane).

Research on endocrine-disrupting compounds reveals the long-lasting effects of exposure to low levels of endocrine disrupters (whereas when carcinogens are removed, the damage is relatively contained) [29]. EDC exposure appears to actually imprint changes on future gene expression (as opposed to gene mutation, which is the effect attributed to classic carcinogens). For example, in 2014 researchers in Chicago found that when male fetuses were exposed to BPA, the chemical reprogrammed their developing prostate and made them more susceptible to prostate diseases later in life [30].

A survey by the Environmental Working Group (EWG) in 2015 found that the average adult uses nine personal care products a day, with 126 unique chemical ingredients [31]. The study, which surveyed more than twenty-three hundred people, found that one in five adults are exposed every day to *all* of the top seven carcinogenic impurities commonly found in personal care products, including formaldehyde, which is listed as a known carcinogen by both the IARC and the National Toxicology Program [2]. The study found that women expose themselves to 168 chemicals every day, about twice as many as men. The average man surveyed said he used five to seven personal care products a day [31]. This can include things like deodorant, toothpaste, shampoo, hair gel, shaving cream, aftershave, and lotion. The average woman uses nine to twelve products. The average teenage girl uses seventeen [32].

Cosmetics such as makeup, moisturizers and hair care products often contain parabens or phthalates, which as we mentioned are endocrine disruptors. Phthalates have been linked to breast cancer, obesity, infertility, and asthma [13, 33]. In 2014 the U.S. Consumer Product Safety Commission recommended that a number of phthalates be banned from children’s toys because studies have connected exposure in rats to abnormalities in the male reproductive tract. The connection is so clear the disorder is called “phthalate syndrome [33].”

Meanwhile, products that promise to protect us from microbes might be doing more harm than good. Many antibacterial soaps, hand sanitizers, and cleaning products contain triclosan. A study in 2013 found that triclosan, like several other chemicals we’ve mentioned, mimics estrogen and could cause the growth of certain types of breast cancer [34].

## The geography of cancer

A cancer cluster is defined by the CDC as “a greater-than-expected number of cancer cases that occurs within a group of people in a geographic area over a limited period of time [35].” These are often work-related, such as the scrotal cancer that was identified in chimney sweeps in eighteenth-century London or the high incidence of osteosarcoma (a type of bone cancer) among the “radium girls,” who painted watch faces with self-luminous paint made with radium in three different factories in the United States in the early twentieth century [36, 37]. There are famous legal cases involving suspected man-made causes of cancers such as the Hinkley, California, tainted water case made famous by Erin Brockovich, who discovered that PG&E (Pacific Gas & Electric) had dumped more than three hundred seventy gallons of chromium-tainted waste water into unlined waste ponds [38]. The groundwater became saturated with chromium 6, a chemical used to inhibit rust. Brockovich and the law firm for which she worked as a legal clerk sued PG&E in 1993 and won what was the largest judgment against a corporate polluter at that time. For the next twenty years, the limited scientific data around this type of water contamination was used as a legal football, even as more cases of cancer and other diseases related to the chromium dump were recorded. Finally, in 2014, after the chromium 6 plume had spread hundreds of miles into other water supplies, this chemical was formally recognized as a known carcinogen [39]. Today, Hinkley, California, is a virtual ghost town.

A more recent high-profile cancer cluster is the one that continues to grow in the aftermath of the September, 11, 2001, attacks on the United States. As of June 2016, the CDC’s World Trade Center Health Program has registered more than fifty-four hundred people diagnosed with cancers thought to have resulted from exposure to carcinogens and pollutants in the aftermath of the attacks [40]. And as time has passed, the rate of reporting has skyrocketed: the latest enrollment *triples* the number of people who had enrolled in 2014, when the registry counted only 1,822 related cancers. Over the last three years, more than fifteen hundred people have been added to this list annually and this number only counts those who have stepped forward to enroll in this federal health program [41]. The hardest hit group is first responders, with 4,692 now receiving health care and medical monitoring through the program. The others are people who lived, worked, or went to school near the World Trade Center. Almost half of the 5,441 enrolled are between the ages of fifty-five and sixty-four.^40^ All totaled, those enrolled in the program report 6,378 separate incidences of cancer, which indicates that some people have been diagnosed with more than one type of cancer since their exposure to toxic dust that included an assortment of toxins, including asbestos, lead, polychlorinated biphenyls (PCBs), polyaromatic hydrocarbons (PAHs), glass fibers, and dioxin to name a few [42].

More troubling are the low-level exposures to carcinogens that affect the whole planet in the air we breathe. A 2017 paper published in the *New England Journal of Medicine* found that U.S. air pollution levels even at concentrations below the current national standards were associated with increased mortality [3]. Though cancer deaths in the United States have fallen by more than 20 percent between 1980 and 2015, an examination of the records from the National Center for Health Statistics has allowed researchers to “map” those regional areas where cancer death rates have not followed this trend [43]. For instance:

- There is an eighty-five-mile-long corridor along the Mississippi River between New Orleans and Baton Rouge, Louisiana, that’s known as “Cancer Alley [44].” This stretch of poorer parishes (counties) is home to more than 150 factories and refineries—and a whole host of pollution-driven diseases, including cancers.
- Along the west Texas border with Mexico, there’s been a huge uptick in the onset of liver cancers, though researches have not yet been able to pinpoint the cause [45].
- Florida—which is home to seventy-seven Superfund sites (environmental hazardous waste sites), thus ranking it sixth in this category in the country—was projected, in 2016, to rank second in the number of new cancer cases [46]. A recent study published in *Statistics and Public Policy* examined cancer onset rates in Florida from 1986 to 2010 and found a strong association between the presence of these toxic sites and the rising rates of cancer [46].

## The uphill battle of environmental protection

To illustrate how slowly environmental reform happens, one only needs to look at the Toxic Substances Control Act of 1976 (the TSCA). The law was designed to regulate the introduction of new chemicals or assess existing chemicals as being “safe” before they are released into the marketplace. However, when the law was enacted in 1976, all existing chemicals were deemed safe for use and were “grandfathered” in. It wasn’t until 2016 that this law was ever updated, and the changes made then—even forty years later—were minimal. In an article published in May 2016, Ken Cook, president and co-founder of the EWG, called out the revised law for doing too little “to protect Americans from chemicals that cause cancer and nervous system disorders, impaired fertility, immune system dysfunction, and a host of other health problems [47].” He made special note of what he calls the “Seven Deadly Poisons” that remain in circulation, despite our knowing (for decades, in some cases) how dangerous they are to the public. These poisons are identified as follows:

*Asbestos: *Despite the direct link between asbestos and lung cancer and other disease being scientifically established, it is still legal and used in the United States even as it is banned in more than fifty other countries [48]. You can still find asbestos in roofing and vinyl materials, brake pads, and other auto parts such as clutches, and it was even recently detected in crayons.

*Formaldehyde: *This naturally occurring substance becomes carcinogenic in high doses and is found in carpets, wood flooring, hair-straightening products, fingernail polish, paints and varnishes, and household cleaning products. Formaldehyde can damage DNA and exposure over time—even in low doses—is known to increase cancer risk [49].

*PFCs: *Perfluorinated chemicals are nonstick, waterproof, and grease resistant and are used in cookware, weatherproof outerwear, and food packaging. Though a class of these chemicals known as C8s (8 indicates the number of carbon atoms) are no longer made in the United States, C6s still are, and are still in use. These have been linked to cancer and thyroid disease, among other health issues [50].

*Fire Retardants: *Chlorinated fire retardants are sprayed onto upholstered furniture and many products made for children, including car seats. They are linked to cancer and hormone disruption [51].

*Vinyl Chloride: *This is used to make PVC plastics and many household products, such as shower curtains. Exposure to airborne PVC affects the nervous system and long-term exposure can cause liver damage [52].

*Bisphenol A (BPA): *This compound can be found in food and beverage containers—and in people, including babies in the womb. In 2015, California took the step of listing BPA as a female reproductive toxicant [53].

*Phthalates: *These compounds make plastics more flexible and are used in PVC plastics, solvents, vinyl flooring, adhesives, and detergents. They are endocrine disrupters and have been linked to diabetes, obesity, and reproductive and thyroid problems [54, 55].

In all of this, it is important that we all understand what we can change and what we cannot when it comes to environmental toxins. We need to collectively become aware of the main known toxins that show up in our air, water, food, and other consumer products (beauty aids, clothing, home-building materials and household goods). For example, most of us have no idea what’s in our water supply: we simply turn on the tap and take a drink. But because of a few horrible events that get national media attention, we’re beginning to take notice. The Flint, Michigan, water crisis gained national attention when the water supply was tainted with dangerous bacteria, factory runoff, and lead [56]. Although many may see this as an isolated event and not relevant to the country as a whole, the EWG in a recent report revealed that an industrial solvent classified as a likely carcinogen (1,4-dioxane), which is also a common impurity in cosmetics and household cleaners, was detected in drinking water supplies for nearly 90 million Americans in forty-five states [57].

Getting clean water seems like it should be a given for most of us, but it’s not as easy as you’d think. Most water bottles made from plastics leech unsafe chemicals like BPA [58]; though many are now BPA-free. While a company may have removed one harmful substance, they may have replaced it with an equally or more harmful substance. For example, BPA has been removed from many products and replaced with bisphenol S (BPS), a chemical that causes the same or worse harm as BPA [59, 60]. A 2013 study by Cheryl Watson at The University of Texas Medical Branch at Galveston found that even small concentrations of BPS can disrupt a cell’s normal functioning, which could potentially lead to the same risks associated with the endocrine disrupting effects as BPA [60].

## Proceed with caution: discussing environmental exposures with patients

When you realize the extent to which we live within a chemical-laden environment, it can make you paranoid. It is likely that most environmental toxins will never be conclusively tied to cancer, but the list of those that have been is growing and we have no reason to believe it will not continue to grow as we become more adept at tracing chemicals and studying their effects on the human body over time. Given the lax regulation of chemicals and the reactionary approach of government regulators, it is up to the consumer to be diligent about reading labels and making healthy choices to limit exposure to chemicals and toxins. It is ideal to encourage patients to adopt the *Precautionary Principle*: until a chemical is found to be harmless, try to not use it. The precautionary principle means that you are maintaining awareness of what you are putting on and in your body and taking steps to avoid exposing yourself unnecessarily to toxins in your household and environment. Encourage patients to start with the simple things, making a clean sweep of their body and house. Patients can examine their exposures from the top of their head to the tips of their toes. When it comes to personal care products, anything that lists on its ingredients “fragrance” includes chemicals that are likely to be endocrine disruptors. Look for products that have ingredients you recognize, without the parabens or phthalates that can mimic hormones in our bodies. Then they go from room to room and look at everything they come in contact with—from the products they use to clean the house to the chairs they sit on and the beds they sleep in. They can slowly clear their body and home as much as they can of potentially dangerous chemicals, and protect their children and their community when possible from the ever-expanding realm of toxic exposure. Good resources for more information on the topic and safer alternatives include websites that are government sponsored (e.g., “.gov”) or that have no commercial interests (e.g., “.org” or “.edu”). Some good websites we recommend include: EWG (ewg.org), Breast Cancer Prevention Partners (bcpp.org), IARC (iarc.fr or monographs.iarc.fr), National Institute of Environmental Health Sciences (niehs.hih.gov), National Cancer Institute (cancer.gov), and the American Cancer Society (cancer.org). It goes without saying that they should avoid too much sun and never ever smoke or use e-cigarettes. Sun and smoke are known carcinogens, so patients need to know about avoiding overexposure to the sun (including tanning beds) and any exposure to tobacco and the chemicals in e-cigarettes.

This better-safe-than-sorry approach is the only way in our current deregulated environment to reduce your contact with known and suspected carcinogens that are present in everything from the fire-resistant couch where you relax every evening to endocrine disruptors in the shampoo and other personal care products you use every morning. Protecting yourself comes down to a simple philosophy—control what you can control, limit your exposure where you can, and then be active in your community when other environmental dangers come to light.

## Financial disclosures

There are no financial disclosures, any conflicts or potential conflicts of interest from any authors.
